# Use of a radial projection to reduce the statistical uncertainty of spot lateral profiles generated by Monte Carlo simulation

**DOI:** 10.1002/acm2.12184

**Published:** 2017-09-18

**Authors:** Xiaoning Ding, Wei Liu, Jiajian Shen, Aman Anand, Joshua B. Stoker, Yanle Hu, Martin Bues

**Affiliations:** ^1^ Department of Radiation Oncology Mayo Clinic Hospital Phoenix AZ USA

**Keywords:** beam modeling data, lateral profile, Monte Carlo simulation, proton spot scanning therapy, statistical uncertainty reduction

## Abstract

Monte Carlo (MC) simulation has been used to generate commissioning data for the beam modeling of treatment planning system (TPS). We have developed a method called radial projection (RP) for postprocessing of MC‐simulation‐generated data. We used the RP method to reduce the statistical uncertainty of the lateral profile of proton pencil beams with axial symmetry. The RP method takes advantage of the axial symmetry of dose distribution to use the mean value of multiple independent scores as the representative score. Using the mean as the representative value rather than any individual score results in substantial reduction in statistical uncertainty. Herein, we present the concept and step‐by‐step implementation of the RP method, as well as show the advantage of the RP method over conventional measurement methods for generating lateral profile. Lateral profiles generated by both methods were compared to demonstrate the uncertainty reduction qualitatively, and standard error comparison was performed to demonstrate the reduction quantitatively. The comparisons showed that statistical uncertainty was reduced substantially by the RP method. Using the RP method to postprocess MC data, the corresponding MC simulation time was reduced by a factor of 10 without quality reduction in the generated result from the MC data. We concluded that the RP method is an effective technique to increase MC simulation efficiency for generating lateral profiles for axially symmetric pencil beams.

## 1 INTRODUCTION

Proton spot scanning has become increasingly popular in radiation therapy because of its superior dosimetry characteristics.^1^ To properly model the dose deposition, treatment planning system (TPS) usually requires a large number of in‐air and in‐water lateral profiles of proton pencil beams as input data.^2^ The dose calculation of proton spot scanning treatment plans relies on the accuracy of these lateral profiles. These profiles generally have a Gaussian distribution except at the low‐dose region where a broad tail is observed.^3^ They can be obtained by measurement^4^ or generated by Monte Carlo (MC) simulation.^5^

Measuring all the lateral profiles are cumbersome because of the extensive amount of data required. To characterize the proton lateral profile accurately, it is required to measure the broad tail down to 0.01% of the central dose.^2^ Obtaining all in‐air and in‐water profiles with satisfied quality demand enormous amounts of resource in terms of proton beam time and physics expertise. As an alternative, generating lateral profiles MC simulation becomes attractive due to the easiness of accessing powerful computational facility.^2^ After being benchmarked against measured data, the MC code can be used to generate the lateral profiles. Another advantage of the MC approach is to make the beam model available in the early stage of commissioning, or even before the start of commissioning if equipment vendor can provide benchmark data beforehand. This provides more time to physicists for commissioning TPS, physicians and dosimetrists to get familiar with proton treatment planning.

MC simulation is by nature a stochastic process. Its outcome inevitably comes with statistical uncertainty. The common practice to reduce the uncertainty is to use variance reduction techniques (VRT), process a large number of particle histories, or increase the scoring volume during simulation.^6^, ^7^ VRT in Monte Carlo calculation can often reduce the computation time required to obtain result with sufficient precision. Types of VRT include energy cutoff, geometry splitting/Russian roulette, and energy splitting/Russian roulette. The application of VRT usually requires care and expert knowledge to choose the appropriate technique(s). Without a good understanding and caution, VRT might actually increase the variance. The approach of using large number of particle histories consumes more computational resource. Usually increasing scoring volume results in lower resolution. However, the proton pencil beam with axial symmetry is an exception. By taking advantage of the axial symmetry, we discovered a method to reduce the statistical uncertainty for computed lateral profile. The method uses the mean value of multiple independent scores as the representative score that can substantially reduce the statistical uncertainty.

## 2 METHODS AND MATERIALS

### 2.A A MC simulation code developed on Geant4 platform

**Figure 1:**
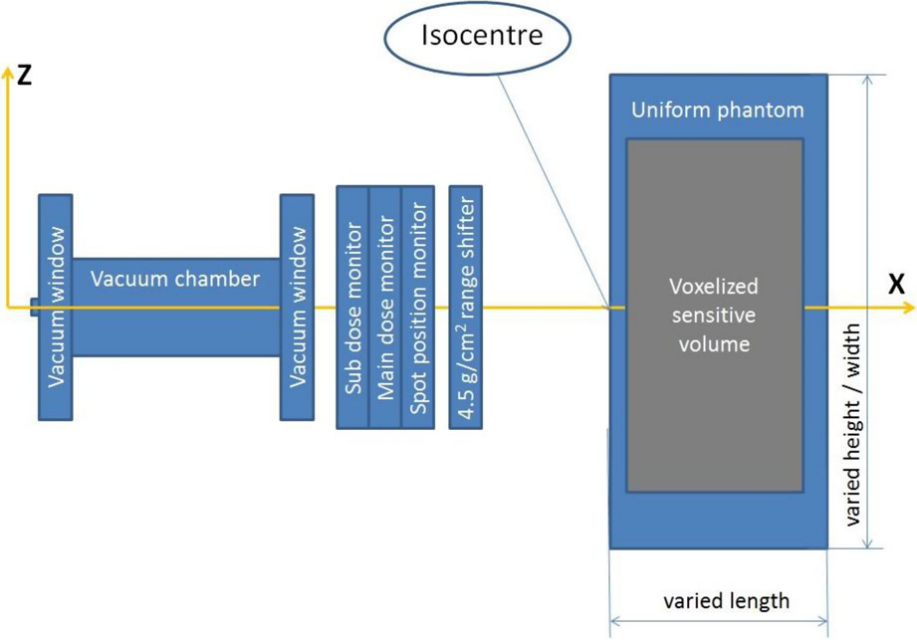
Components of the proton scanning beam nozzle. The beam direction was parallel to x axis, propagating from left to right. The nozzle contains vacuum chamber with titanium windows, main and sub dose monitors, and optional range shifter of 4.5 g/cm^2^ positioned at the distal end of the nozzle. A uniform phantom was placed at the end of beam line.

The MC simulation code, developed on a Geant4 platform, models the spot scanning nozzle for our proton therapy center. Geant4 is a toolkit for simulating the transportation of particles through media.^8^ It provides a complete set of functionality modules for developing MC simulation codes for many particle‐physics‐related applications, including the clinical applications of proton and ion beams. The Geant4 application is implemented in the C++ programming language, and therefore demands significant resources from medical physicists and computer engineers. For simplicity and expediency, we developed the MC code for our project by just adding one new module to an existing Geant4 example *Hadrontherapy,* 9 which is contained in the official Geant4 distribution (http://geant4.web.cern.ch/geant4/). The new module describes the geometries and material compositions of the proton scanning beam nozzle, according to the design configuration provided by the nozzle manufacturer (Fig. 1). The geometry module contained a vacuum drift chamber with titanium windows, two plane‐parallel ionization chambers (main and sub dose monitors), and a spot position monitor that consisted of a multiwire ionization chamber. An optional range shifter of 4.5 g/cm^2^ can be placed at the distal end of the nozzle. A cuboid‐shaped uniform phantom was placed at the end of beam line for the purpose of scoring dose deposition. Inside the phantom, a cuboid‐shaped sensitive volume was voxelized. At the end of each MC run, the dose deposition in each voxel is collected and saved to a file in ASCII format. The proton source is modeled by free parameters including energy, momentum, and spatial distribution. The approach of free parameters has been proven to be a good solution when handling proton source with large uncertainty and additional uncertainty introduced by the MC algorithms.^5^ A Gaussian shape in the spatial distribution was used to model the cross section of proton source. Since the proton source, nozzle components and water phantom can all be treated as axial symmetry, the dose distribution in the water phantom should be axially symmetrical.

Ideally for this axial geometry, ring tally would be the default choice to take advantage from symmetrical geometry for scoring lateral profile. Gate^10^ and Topas,^11^ that are based on Geant4, provide the possibility to model ring geometry and scoring. We decided to develop MC code directly on the Geant4 toolkit to maximize the benefit that Geant4 can provide. The Geant4 example code *Hadrontherapy* comes with the implementation of cuboid scoring volume, using 3D Cartesian coordinate system. The implementation of ring tally requires cylindrical or cylindrical‐like coordinate system to score dose deposition. Applying a cylindrical system would require completely rewriting the dose‐scoring module, requiring extra code development. Another possible approach would be to build a cylindrical‐like scoring tally system on top of the 3D‐Cartesian coordinate system, as follows:

1. Define multiple flat layers in the phantom as sensitive volumes.
2. For each flat layer, define a histogram for radius in bins of an arbitrary large number.
3. During MC simulation for every energy deposition at voxel (x, y) in any given layer, find the corresponding bin by radius $r=\sqrt{{\left(x-x_{0}\right)}^{2}+{\left(y-y_{0}\right)}^{2}}$ and fill the corresponding histogram, assuming the beam center is at (*x*_0_, *y*_0_).
4. The resultant histogram (that is, total energy) divided by the corresponding bin mass shows the dose profile as a function of radius.

Although the cylindrical‐like approach allows the lateral profiles to be obtained directly from MC simulation, it still requires some code development. Also the work to re‐bin data during MC simulation, as stated in steps (2) to (4) above, is an extra burden to computational resource. To avoid any new code development or the downgrade of the MC performance, we used a completely different approach. We take the dose output data from the MC simulation, which is in 3D‐grid format, radially projected all voxel scores according to the distance to the beam center, calculated the mean value of multiple independent scores with the same radius, and used the mean as representative value for the radius. This approach did not require any new code development, nor did it add extra burden to MC simulation. The method is called as radial projection method, and described it in the next section.

### 2.B Radial projection method

The radial projection (RP) method assumes a pencil beam with axial symmetry impacting perpendicularly on a plane detector. The workflow for the RP method is as follows.

1. Voxelize the cuboid scoring volume using cuboid voxels.
2. Align pencil beams to be perpendicular to the scoring volume surface, with the beam center pointing to the center voxel on the surface.
3. Project all voxel scores to an axis based on the radial projection distance of the voxels.
4. Calculate the mean of the scores with the same radial projection distance, and use the mean as the representative score for the voxels with the same radial projection distance.

**Figure 2:**
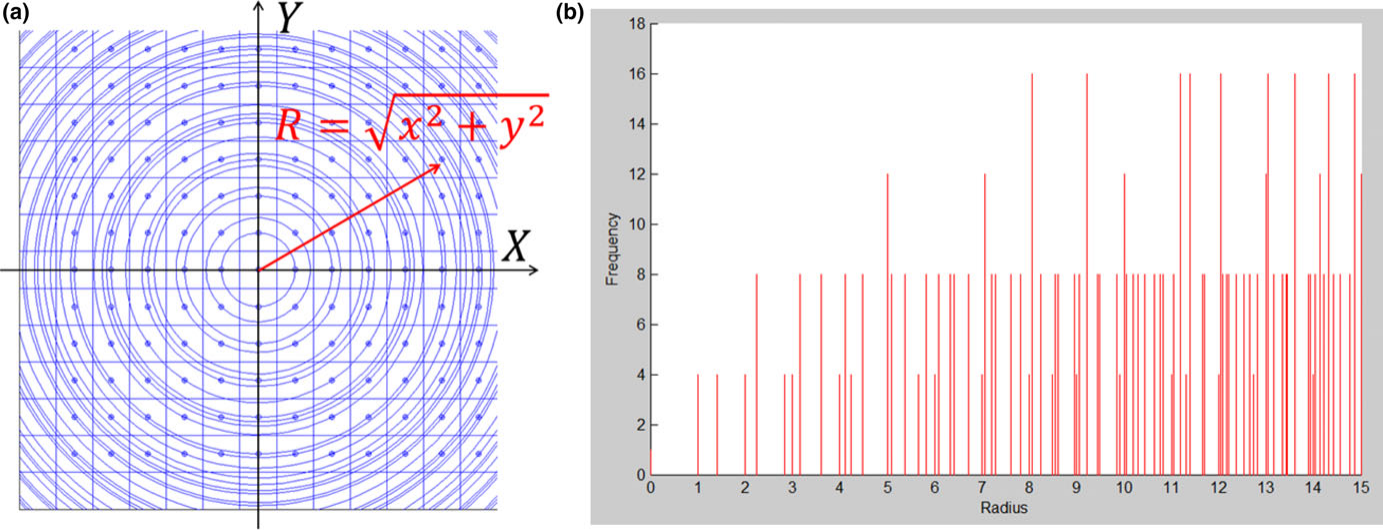
(a) Projecting voxels on a square plane to a one‐dimensional axis and (b) the number of voxels (frequency) with the same radial distance. The pencil beam center is defined as the origin of coordinates. The voxel centers on any given circle have the same radial distance.

Figure 2 illustrates the concept of the RP method. Figure 2(a) shows a square plane voxelized by a unit square. The centers of voxels with the same radius are connected by circles, that is, the voxels with their centers on any given circles have same radius. Figure 2(b) depicts the voxel frequency vs the radial projection distance. As shown in the Fig. 2(b), there are four or more voxels except at the origin (0, 0). For example, there are four voxels at the radius of 1.414, which corresponds to the voxels at (1, 1), (−1, 1), (−1, −1), and (1, −1). There are 12 voxels at the radius of 5, which correspond to the voxels at (5,0), (4,3), (3,4), (0,5), (−3,4), (−4,3), (−5,0), (−4, −3), (−3, −4), (0, −5), (3, −4), and (4, −3). Rather than use any individual score, the mean is taken as the representative score for the voxels at this radius. It is well known that the statistical uncertainty is approximately reduced by the square root of the number of histories, N. Therefore the statistical uncertainty is halved at the radius 1.414 and is reduced to about 29% ($1/\sqrt{12}=28.87\%$) at the radius of 5. Another nice feature of the RP method is the number of voxels with the same radial projection distance becomes larger as the radius increases. As shown in the right panel of Fig. 2, there are four voxels within the radius between 1.0 and 2.0. However for the radius between 5.0 and 6.0 there are 28 voxels.

The proton dose deposition is largest in the area near the pencil beam center. The score of dose deposition near the center usually has better statistics. For the voxels far away from the beam center, the score is more fluctuated due to less particle history. However as shown in Fig. 2(b), the score density becomes nearly continuous for larger radii. One can take advantage of it to further reduce the data fluctuation. In this study, we used radius bins of variable sizes to further average the mean scores within a small radius interval. The mean from the scores with the same radius is termed *point average*, while the mean from the scores within a radius bin is termed *interval average*. A point on the radius axis, called *boundary* (*R*_0_), is used to divide the radius axis into high‐dose region and low‐dose region. In this study, we applied the point average to the high‐dose regions (*r* < *R*_0_), and the interval average to the low‐dose regions (*r* ≥ *R*_0_). The mean score of radius interval $\bar{S}\;$(as defined by eq. (1)) and mean radius (as defined by eq. (2)) were used as the representative value for any given radius interval.

(1) $$\bar{S}=\frac{\sum {\left(scoreofradius\right)}_{i}}{\sum^{i}}$$

(2) $$\bar{R}=\frac{\sum {\left(radius\right)}_{i}}{\sum^{i}}$$

**Figure 3:**
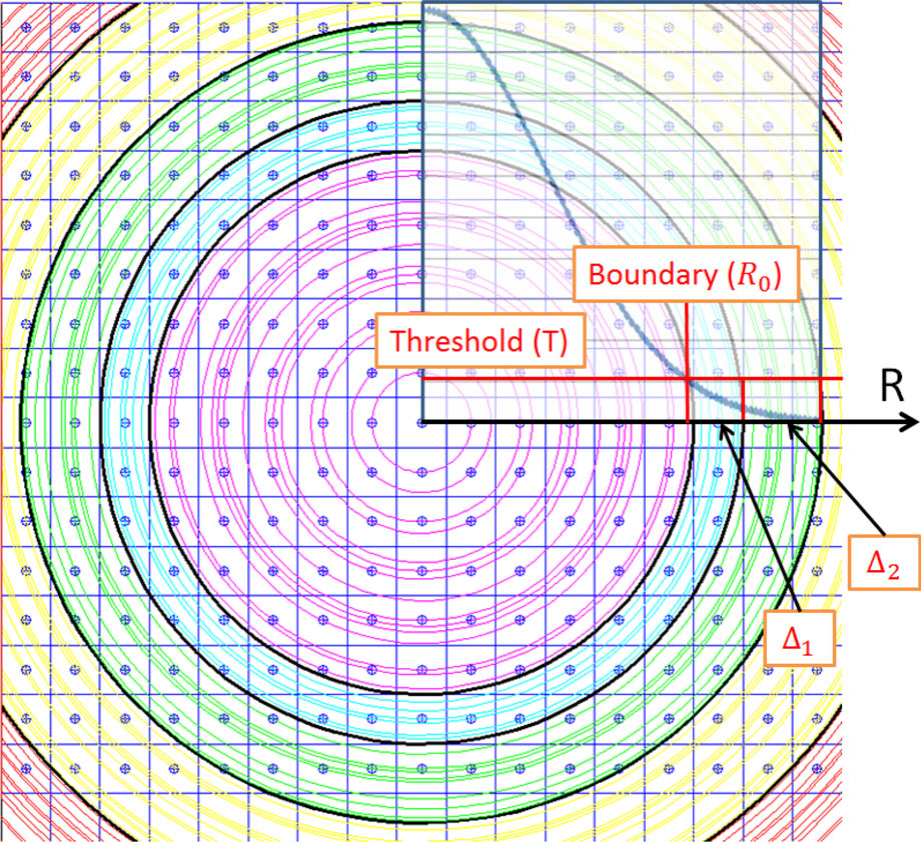
Lateral dose decreases gradually with increasing radius. The boundary (*R*_0_) is the first point at the radius axis whose corresponding point average dose is either equal to or less than the value of threshold (T). Point average was performed in the high‐dose region (*r* < *R*_0_). Interval average was performed in the low‐dose region (*r* ≥ *R*_0_). Δ_1_ represented by cyan ring is the first radius interval and Δ_2_ represented by a green ring is the second radius interval.

We introduced a parameter called *threshold* (T) to define the boundary and the width of radius intervals (Δ*s*). Figure 3 illustrates the concepts of threshold and boundary. As shown in Fig. 3, the lateral dose decreases gradually with increasing radius. The boundary (*R*_0_) is defined as the first radius point whose corresponding point average dose is either equal to or less than the value of threshold (T). In the high‐dose region (*r* ≤ *R*_0_) represented by the magenta circle lines, point average was performed to obtain the mean score. After the radius passes the boundary, interval average was performed. The width of any radius interval Δ is wide enough to contain enough radius points that the sum of point averages is either equal to or larger than the value of threshold (T). As shown in Fig. 3, the 1st interval is Δ_1_ represented by a cyan ring and the 2^nd^ interval is Δ_2_ represented by a green ring.

### 2.C Relative standard error

In this study, we use the relative standard error (RSE) to quantify the statistical uncertainty for the MC‐generated data. The RSE is defined as eq. (3).

(3) $$RSD=\frac{\sqrt{\sum_{i}^{n}{\left(x_{i}-\bar{x}\right)}^{2}/\left(n\times \left(n-1\right)\right)}}{\bar{x}}\times 100\%$$

where the x_i_ is the individual score of the voxels that correspond to the given radius, and $\bar{x}\;$ is the mean of all scores in the sample set.

## 3 RESULTS

### 3.A Statistical uncertainty reduction by the RP method

**Figure 4:**
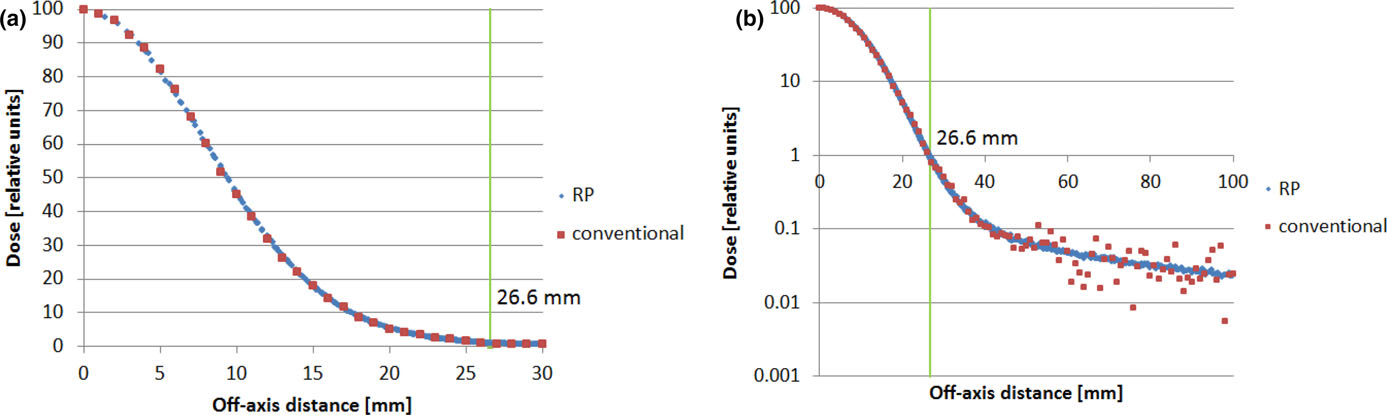
Lateral in‐air dose profiles of proton beam 144.8 MeV at the Isocenter with 2 × 10^7^ proton histories (a) in linear scale and (b) in semi‐log scale. The green vertical lines represent the boundary at the radius of 26.6 mm (radial projection method, blue; conventional method, red). A range shifter of 4.5 g/cm^2^ was placed 42.5 cm upstream from the isocenter.

Figure 4 shows the profile comparison between the conventional and the RP methods for an in‐air lateral dose profile of a proton beam of 144.8 MeV at the isocenter. A range shifter of 4.5 g/cm^2^ was placed 42.5 cm upstream from the isocenter. The voxel size of the sensitive volume was 1 × 1 × 1 mm^3^, and 2 × 10^7^ protons were used in the MC simulation. We defined the conventional method as scores in the direction out from the pencil beam center. Figure 4(a) depicts the profile in linear scale for the high‐dose region, and Fig. 4(b), the semi‐log scale. As shown by other investigators, the lateral profiles of the proton pencil beam usually extend out greatly in low‐dose regions, that is, with a broad low‐dose tail.^3^, ^5^, ^12^ This low‐dose tail can be displayed more clearly using a semi‐log scale. The boundary corresponding to 1% of the peak dose is 26.6 mm, as represented by the green vertical lines. The point average was used in the high‐dose region (off‐axis distance <26.6 mm), and the interval average was used in the low‐dose region (off‐axis distance ≥26.6 mm).

**Figure 5:**
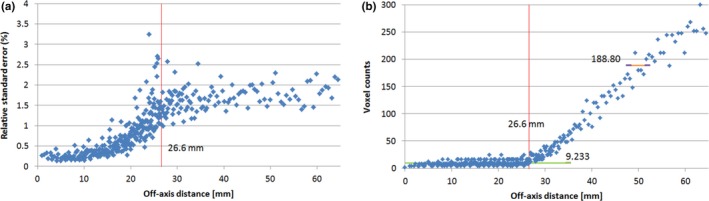
Relative standard error (a) and voxel counts (b) of proton beam 144.8 MeV at Isocenter plane. The vertical red lines represent the boundary at radius 26.6 mm. In (b), the horizontal green line represents the average voxel count (9.233) in the region of off‐axis distance <26.6 mm. The horizontal brown line represents the average voxel count (188.80) at radius 50 mm.

We applied the RSE analysis to the same dataset. Figure 5 shows the variation in RSE as a function of the off‐axis distance [Fig. 5(a)] and the corresponding number of voxels [Fig. 5(b)]. As shown in Fig. 5(a), the RSE value increases gradually as the off‐axis distance approaches the boundary at radius 26.6 mm and stabilizes after passing the boundary. The average number of voxels per radial position was 9.233 in the region of the off‐axis‐distance <26.6 mm, as represented by the horizontal green line in Fig. 4(b). On average, the data fluctuation is suppressed to about one‐third of its original value. The number of voxels in the low‐dose tail region (that is, off‐axis‐distance ≥26.6 mm) is much higher. For example, the average number of voxels at a radius position of 50 mm is 188.80, as represented by the horizontal brown line in Fig. 5(b). The corresponding noise is suppressed to 7.27% of its original value. Note that the interval average is used at this radial position of 50 mm, while the point average is used in the high‐dose region. The interval average at the radius of 50 mm is about 4.5 times more effective ($32.93\%/7.27\%=4.53$) in noise reduction than the point average in the high‐dose region.

### 3.B MC simulation time reduction

**Figure 6:**
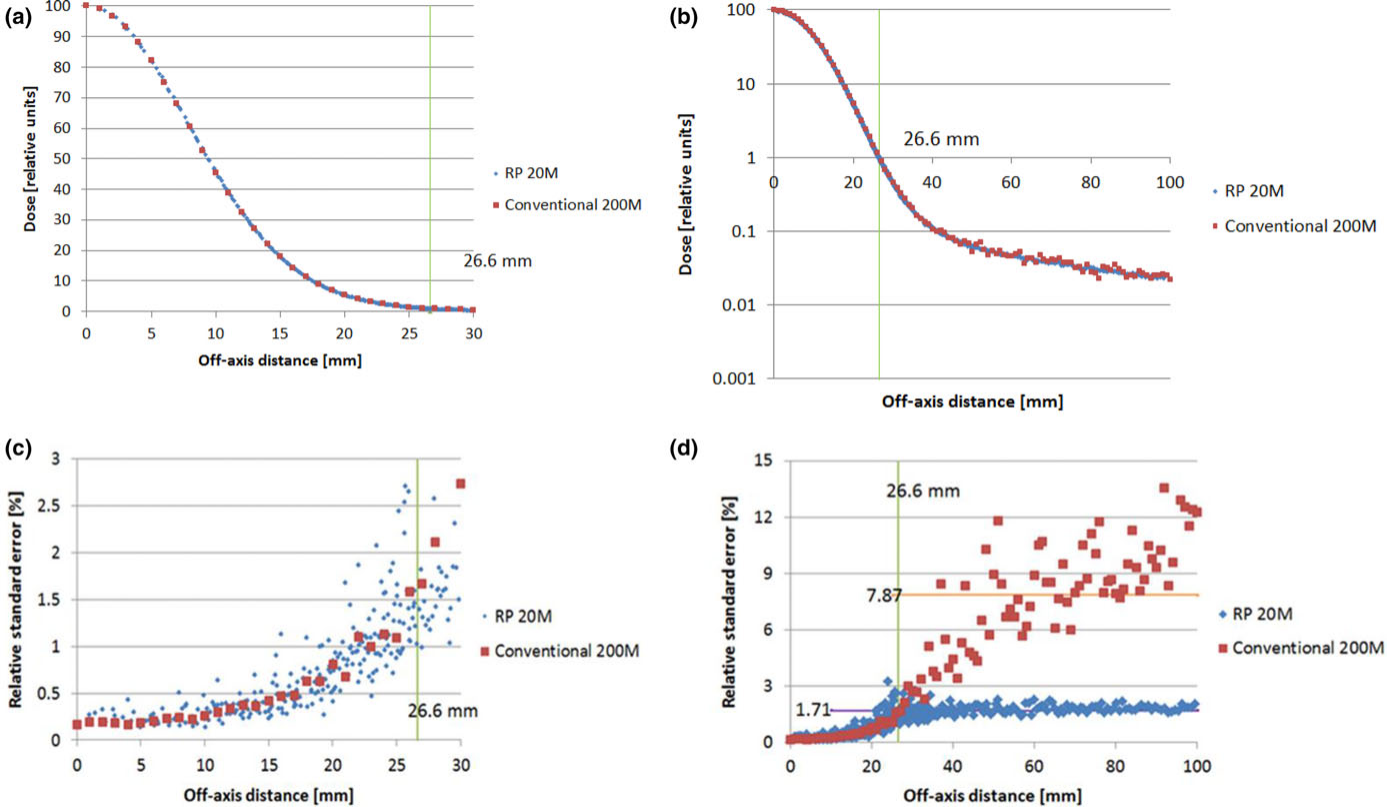
Upper panels (a) and (b) show the in‐air lateral profiles generated by the radial projection (RP) method (20 million protons, blue) and the conventional method (200 million protons, red): (a) linear scale; (b) semi‐log scale. Corresponding relative standard errors (RSE) are shown in the lower panels, with off‐axis distance up to 30 mm (c) and up to 100 mm (d). The energy of the proton pencil beam was 144.8 MeV. A range shifter of 4.5 g/cm^2^ was placed 42.5 cm upstream from the isocenter. The raw data from the Monte Carlo simulation were taken at the isocenter plane (RP method, blue; conventional method, red).

Because the RP method can reduce statistical uncertainty, it was expected that the RP method would reduce the number of required histories, as well as the MC simulation time. We ran the MC simulation twice for the same condition (144.8 MeV and 4.5 g/cm^2^ were placed 42.5 cm upstream from the isocenter). In the first run, 2 × 10^8^ protons were used; in the second run, 2 × 10^7^ protons were used: 10 times less than that in the first run. The conventional method was used to obtain profiles from the first run's output, and the RP method was used to obtain profiles from the second run's output. Figures 6(a) and 6(b) show the profile comparison and Figs. 6(c) and 6(d), the RSE comparison. The RP method brought in more data points (blue). In the high‐dose region (off‐axis distance <26.6 mm), the profile generated by the RP method contained 261 radius points, and each point had 9.233 scoring voxels on average. For the same dose region, there were 27 radius points (red), and each radius point had 1 scoring voxel by the conventional method. The lateral profile by the RP method was smoother, especially in the low‐dose tail region. The RSE by the RP method was only 1.71%, represented by the purple line in Fig. 6(d). The RSE by the conventional method was 7.87%, represented by the orange line which is 4.6 times greater than the RSE by the RP method.

### 3.C Comparison between MC calculations and measurements

**Figure 7:**
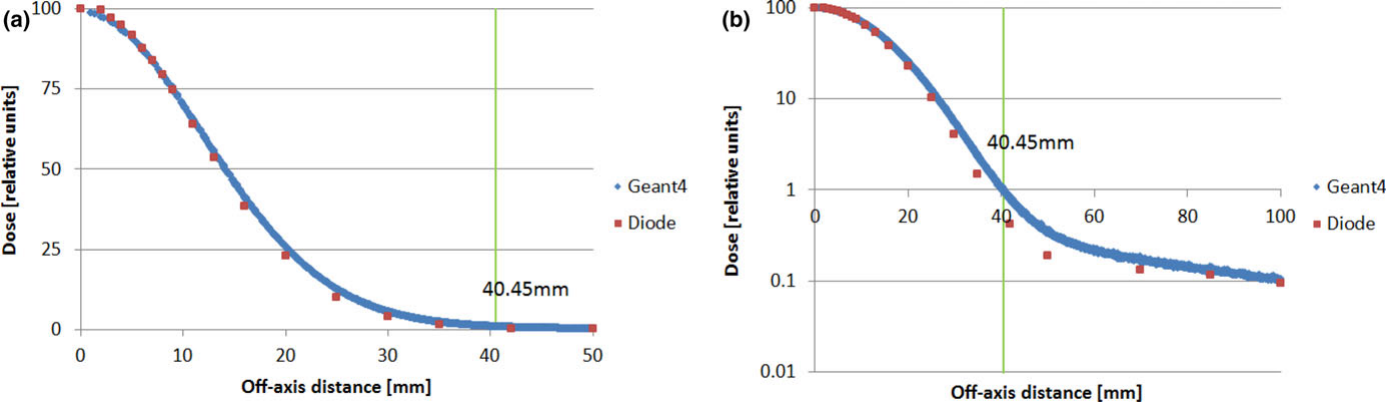
Comparison of an in‐air lateral dose profile of a proton beam of 90.1 MeV at the isocenter, with a range shifter of 4.5 g/cm^2^ placed 42.5 cm upstream from the isocenter: (a) linear scale; (b) semi‐log scale (Monte Carlo calculated profile, blue; diode measurement, red). 8 × 10^7^ protons is used in the simulation.

**Figure 8:**
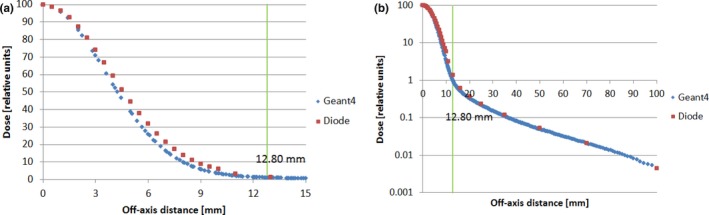
Comparison of in‐water lateral dose profile for the proton beam of 228.8 MeV at a depth of 210.5 mm: (a) linear scale; (b) semi‐log scale (Monte Carlo calculated profile, blue; diode measurement, red). 4.8 × 10^7^ protons is used in the simulation.

**Figure 9:**
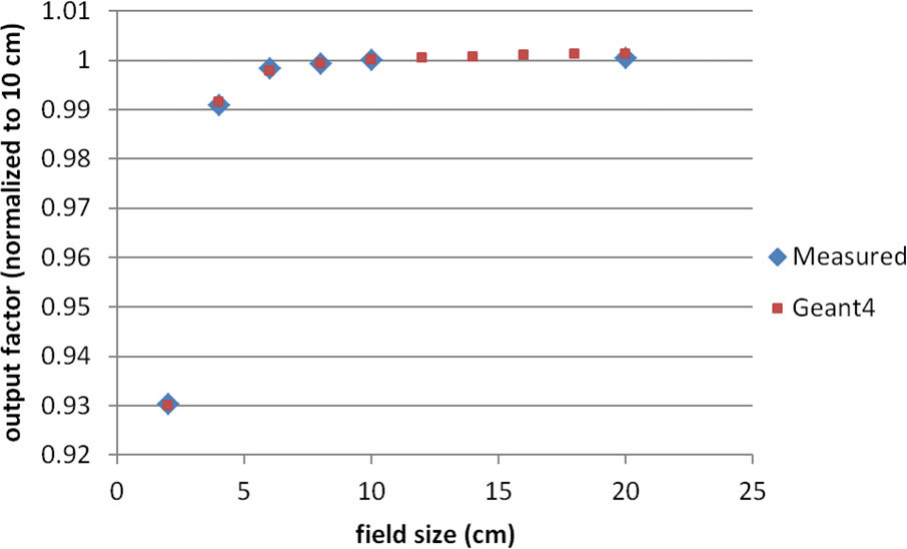
Comparison of output factor (OF) for the proton beam of 100.7 MeV at water depth of 50 mm. (Monte Carlo calculated OF, red; measurement, blue).

The lateral dose profiles, generated by postprocessing MC data by the RP method, were used to compare with measurements in two different ways. First, we directly compared MC‐generated profiles with measured profiles. Second, we compared the derived output factor (OF) of square field from MC‐generated profiles with the measured OF. The MC calculations agreed reasonably well with the measurements in both comparisons. It should be pointed out that the good agreement demands more than just a data‐noise‐reduction technique, such as the RP method. It at least requires properly model spot scanning nozzle with accurate geometry and applicable physics process, as well as carefully perform measurement.^4^, ^5^ An accurate measurement of proton lateral profile is challenging, because the low‐dose tail is usually below 0.1% of central dose.^3^ However, the application of the RP method did make contribution to improve the agreement. Figures 7 and 8 show two examples of direct profile comparisons: an in‐air (Fig. 7) and an in‐water (Fig. 8) lateral dose profile. In both cases, measured data were obtained by semiconductor diodes (PTW, Diode PR TN60020). In Fig. 7, data are shown for a proton pencil beam of 90.1 MeV at the isocenter plane and a range shifter of 4.5 g/cm^2^ upstream from the isocenter. In Fig. 8, data are shown for a proton pencil beam of 228.8 MeV at a water depth of 210.5 mm. As shown in Figs. 7 and 8, the MC‐calculated dose profiles agree reasonably well with the measured profiles. For the OF comparison, more than 50 MC‐generated output factors were used to compare with the corresponding measurements, including seven proton energies, various field size from 2 to 20 cm and various depths from 20% to 80% of proton range.^13^ Figure 9 shows an example of the OF comparisons for the proton beam of 100.7 MeV at water depth of 50 mm. The MC‐calculated output factors agreed well with the measured ones from a small field (2 × 2 cm) to a large field (20 × 20 cm).

## 4 DISCUSSION

The application of the RP method substantially reduced the statistical uncertainty of spot lateral profiles generated by MC simulation for an axial symmetric pencil beam. The RP method has several advantages over the conventional method (a) it can generate a lateral profile of superior quality with the same particle history as shown in Figs. 4(a) and 4(b); or (b) a lateral profile of the same quality with 10 times less particle history, as shown in Fig. 6. Therefore, the efficiency of generating spot lateral profiles can be increased substantially by the RP method. Two key factors improve the efficiency. First, the RP method provides more independent scores at any given radius position. Second, the RP method provides more independent scores for a given radius interval. The quality improvement is more prominent in the low‐dose tail region. A lateral dose profile with spot shapes down to a level of 10^−4^ can be easily generated with 10^8^ proton histories, as long as the full width at half maximum of the lateral profiles is less than 15 mm. Other investigators have shown, which was confirmed by our experience, that lateral dose profiles with spot shapes to the 10^−4^ level are needed to model the proton pencil beam properly.^2^ The merit of the RP method is in taking more independent scores without sacrificing spatial resolution. Indeed, the RP method uses information from every voxel in the sensitive volume to construct the lateral profile. In comparison, the conventional method uses information from a small portion of voxels. Therefore, the superior result achieved with the RP method is not unexpected.

The RP method obtains mean values from the independent scores at the same radius position (point average) or within a small radius interval (interval average). Essentially, it works in a manner like constructing ring tallies from cuboid tallies. The signal‐to‐noise ratio of a ring tally is obviously higher than that of any single cuboid tally. Therefore, applying the RP method to a cuboid tally offers three additional benefits. First, the cuboid tally is much more flexible than a ring tally because it can score any kind of dose distribution regardless of whether it is symmetrical or nonsymmetrical. Second, the RP method dose not re‐bin dose data during the MC simulation as the proposed cylindrical‐like approach (see 2.A, Geant4 MC Simulation Code). Instead, the re‐binning is performed during the process of data postprocessing, which adds no extra burden to the MC simulation. Third, the Geant4 example *Hadrontherapy* comes with a validated implementation of a cuboid tally. It does not require any additional code development because the RP method takes the output directly from cuboid tallies.

Boundary is a parameter to define the dividing point between high‐dose region and low‐dose region, as well as the width of radius interval. The boundary is expected to shift left and the width of radius width to be increased when the value of threshold is increased. The optimal value for the threshold depends on the shape of lateral profile and total number of primary particles (primary protons in this study). It is a trial‐and‐error process to determine the optimal value of the threshold for a given lateral profile. In this study, the typical threshold value varies between 0.1% and 1.0%. With increasing value of threshold, the lateral profile becomes smoother in the low‐dose region. However, it results in less data points and might potentially introduce distortion if the threshold is too high.

There is a limitation to the application of the RP method. The RP method assumes the dose distribution is axially symmetrical, and error would be introduced when the distribution were not exactly axially symmetrical. The proton pencil beam is usually highly unsymmetrical at the entrance of the spot scanning nozzle. Because of the interaction of the proton particles with nuclei or electrons present in the nozzle's components and air, it becomes more symmetrical gradually down the beam path. In our facility, the cross section of the proton pencil beam has an elliptical shape at the isocenter. The corresponding ratio of the major axis over the minor axis varies from 1.05 to 1.15 with an average of 1.09. However the elliptical shape dies away gradually once proton beam enters to water. We ran a preliminary MC simulation to quantify the decrease in the elliptical shape in water tank. The result shows the difference of major axis to minor axis is approximately reduced to its half whenever proton beam goes through 10 cm water. During the acceptance and commissioning, we have not observed lateral‐profile asymmetry in water tang measurement. Also because the commercial TPS (Eclipse 13.6, Varian Medical Systems, Palo Alto, CA, USA) used in our facility cannot model how the spot ellipticity changes with gantry rotation, we have to treat the pencil beams with axially symmetry, whether or not the range shifter was inserted. The potential error introduced by assumption of this axial symmetry has been also evaluated during commissioning and patient‐specific quality assurance. No noticeable discrepancy has been found thus far.

## 5 CONCLUSION

Herein, we described the concept of the RP method and how it can be implemented, and showed how the results of MC simulation can be improved using the RP method. The RP method is an effective technique for increasing the efficiency of MC simulation in generating spot lateral profiles for axial pencil beams.

## CONFLICT OF INTEREST

No conflict of interest exists in relation to this article.

## References

[1] Lomax AJ , Bortfeld T , Goitein G , et al. A treatment planning inter‐comparison of proton and intensity modulated photon radiotherapy. Radiother Oncol. 1999;51:257‐271. 10435821 10.1016/s0167-8140(99)00036-5

[2] Zhu XR , Poenisch F , Lii M , et al. Commissioning dose computation models for spot scanning proton beams in water for a commercially available treatment planning system. Med Phys. 2013;40:041723. 23556893 10.1118/1.4798229 PMC3631269

[3] Lin L , Ainsley CG , Solberg TD , McDonough JE . Experimental characterization of two‐dimensional spot profiles for two proton pencil beam scanning nozzles. Phys Med Biol. 2014;59:493‐504. 24374943 10.1088/0031-9155/59/2/493

[4] Gillin MT , Sahoo N , Bues M , et al. Commissioning of the discrete spot scanning proton beam delivery system at the University of Texas M.D. Anderson Cancer Center, Proton Therapy Center, Houston. Med Phys. 2010;37:154‐163. 20175477 10.1118/1.3259742 PMC11078095

[5] Sawakuchi GO , Mirkovic D , Perles LA , et al. An MCNPX Monte Carlo model of a discrete spot scanning proton beam therapy nozzle. Med Phys. 2010;37:4960‐4970. 20964215 10.1118/1.3476458 PMC2941520

[6] Kawrakow I , Rogers DWO , Walters BRB . Large efficiency improvements in BEAMnrc using directional bremsstrahlung splitting. Med Phys. 2004;31:2883‐2898. 15543798 10.1118/1.1788912

[7] Rogers DWO , Faddegon BA , Ding GX , Ma CM , We J , Mackie TR . Beam ‐ a Monte‐Carlo code to simulate radiotherapy treatment units. Med Phys. 1995;22:503‐524. 7643786 10.1118/1.597552

[8] Allison J , Amako K , Apostolakis J , et al. Geant4 developments and applications. IEEE Trans Nucl Sci. 2006;53:270‐278.

[9] Cirrone GAP , Cuttone G , Mazzaglia SE , et al. Hadrontherapy: a Geant4‐based tool for proton/ion‐therapy studies. Progress Nucl Sci Technol. 2011;2:207‐212.

[10] Jan S , Benoit D , Becheva E , et al. GATE V6: a major enhancement of the GATE simulation platform enabling modelling of CT and radiotherapy. Phys Med Biol. 2011;56:881‐901. 21248393 10.1088/0031-9155/56/4/001

[11] Perl J , Shin J , Schumann J , Faddegon B , Paganetti H . TOPAS: an innovative proton Monte Carlo platform for research and clinical applications. Med Phys. 2012;39:6818‐6837. 23127075 10.1118/1.4758060 PMC3493036

[12] Clasie B , Depauw N , Fransen M , et al. Golden beam data for proton pencil‐beam scanning. Phys Med Biol. 2012;57:1147‐1158. 22330090 10.1088/0031-9155/57/5/1147 PMC3387676

[13] Shen J , Liu W , Stoker J , et al. An efficient method to determine double Gaussian fluence parameters in the eclipse proton pencil beam model. Med Phys. 2016;43:6544. 27908162 10.1118/1.4967485 PMC6961730

